# Real‐world incidence and clinical characteristics of intraocular inflammation following intravitreal faricimab therapy: A safety study

**DOI:** 10.1111/aos.70161

**Published:** 2026-05-16

**Authors:** Miklos Schneider, Leyla Turan, Farah Arif, Jakob Bjerager, Oliver Niels Klefter, Carsten Faber, Yousif Subhi, Javad Nouri Hajari

**Affiliations:** ^1^ Department of Ophthalmology Rigshospitalet Glostrup Denmark; ^2^ Department of Clinical Medicine University of Copenhagen Copenhagen Denmark; ^3^ Department of Ophthalmology Semmelweis University Budapest Hungary; ^4^ Department of Clinical Research University of Southern Denmark Odense Denmark

**Keywords:** adverse event, anti‐VEGF, faricimab, intraocular inflammation, intravitreal injection

## Abstract

**Purpose:**

To estimate the real‐world incidence of intraocular inflammation (IOI) following intravitreal faricimab injections at a large, highly specialized tertiary centre.

**Methods:**

Single‐centre, retrospective cohort study with chart review using an electronic injection database and electronic medical records from November 2023 to August 2024.

**Results:**

The study included 6054 patients, 7510 eyes and 31 393 injections. During the inclusion period, 42 eyes of 40 patients developed IOI, resulting in 48 IOI events. The incidence of IOI was 0.66% per patient, 0.56% per eye and 0.13% per injection. IOI occurred after a median of 4 faricimab injections (IQR: 2, range: 1–9) and a median of 8.5 days (IQR: 20, range: 0–37) after the last injection. The median disease duration (time from diagnosis to resolution) was 32 days (IQR: 46, range: 6–144). Eighty per cent of patients who developed IOI were females, and females had 2.42–2.66 times higher hazard of developing IOI than males. Most IOI cases were anterior or anterior + intermediate uveitis (47.9% and 33.3%, respectively). All panuveitis cases (*n* = 6) underwent vitrectomy, and one had a positive culture, yielding a 0.003% per‐injection incidence of culture‐positive endophthalmitis. Cases rechallenged with faricimab following IOI (*n* = 13) had a relapse in 46.15%. The change in median best‐corrected visual acuity between before and after IOI events was not statistically significant.

**Conclusions:**

Faricimab demonstrated an acceptable safety profile in our study. The incidence of IOI events was consistent with those observed in other real‐world reports. The IOI events showed a female predominance, varied presentations and durations, but generally had favourable outcomes.

**Study Registration:**

ClinicalTrials.gov registration number: NCT06902207, Date of registration: 15/03/2025 retrospectively registered.

## 1 INTRODUCTION

The introduction of anti‐vascular endothelial growth factor (VEGF) therapies has significantly improved the prognosis for patients with retinal diseases such as age‐related macular degeneration (AMD), diabetic macular oedema (DME) and retinal vein occlusion (RVO) (Hang et al., 2023). These treatments have set a new standard of care by targeting the VEGF pathway, which plays a crucial role in disease progression and its vision‐threatening complications.

Faricimab (Vabysmo®, Roche, Basel, Switzerland), a novel pharmaceutical agent, is a promising treatment for AMD, DME and RVO due to its dual mechanism of action, targeting both the VEGF and angiopoietin‐2 (Ang‐2) pathways, offering potential benefits in improving visual outcomes and reducing treatment burdens (Butler et al., 2025; Khanani et al., 2020). The phase 3 TENAYA and LUCERNE clinical trials have demonstrated their efficacy and safety profile (Heier et al., 2022), leading to regulatory approval (European Medicines Agency, 2022; Shirley, 2022) and increasing use in clinical practice (Abdalla et al., 2025; Borras‐Blasco et al., 2025). However, as with any new therapeutic agent, there is a critical need to monitor and evaluate real‐world safety data to complement and validate clinical trial findings.

Intraocular inflammation (IOI), which is an important adverse effect of intravitreal anti‐VEGF injections, is of particular interest as it can vary from a mild transient reaction to a potentially vision‐threatening outcome (Anderson et al., 2021; Fine et al., 2015).

The incidence and type of IOI in a real‐world setting could potentially differ from clinical trial results due to differences in patient populations, injection techniques and clinical settings.

The treatment and follow‐up of these adverse events require considerable resources, as these patients need frequent follow‐ups and continuous treatment until the IOI is resolved, and sometimes surgical interventions are necessary. Determining the course and functional outcomes of such adverse events is also crucial for setting patient expectations and for being able to convey accurate patient information.

The purpose of this study was to estimate the real‐world incidence of IOI following intravitreal faricimab injections.

## 2 METHODS

### 2.1 Study design

This retrospective single‐centre cohort study and chart review was carried out at the Department of Ophthalmology, Rigshospitalet, Glostrup, Denmark, the largest highly specialized tertiary centre in the Nordic countries.

### 2.2 Ethical approval and consent

The research and data handling were conducted in compliance with local laws and GDPR, the tenets of the Declaration of Helsinki and its amendments. The Regional Health Research Ethics Committees in the Capital Region in Denmark waived the need for ethical approval of the project (no. F‐24041134). The Research and Health Legal Departments of the Capital Region of Denmark approved the patient confidentiality and data flow protocol of the project and waived the need for informed consent (approval no.: p‐2024‐16 902, date of decision: 04/11/2024). The study protocol was registered at ClinicalTrials.gov, registration number: NCT06902207. We followed the STROBE reporting guidelines (von Elm et al., 2007).

### 2.3 Patient selection, inclusion and exclusion criteria

In this study, we included all patients 18 years of age or older who received at least one intravitreal faricimab injection at our centre between 1 November 2023 and 31 August 2024. We enforced no further restrictions, neither with regard to diagnosis nor to previous anti‐VEGF treatment.

Treatments and follow‐ups were performed under routine clinical care conditions, following in‐house standards and local guidelines.

### 2.4 Procedure and follow‐ups

During the selected period, patients received intravitreal faricimab injections given by trained injection nurses or ophthalmology residents. The injections were either pre‐prepared by the hospital pharmacy as ready‐to‐use prefilled syringes (VitreJect® Ophthalmic Syringe LL LDS, 1 mL; OcuJect LLC, CA, USA), multiple doses from a single vial in sterile packaging, or drawn in the injection rooms from original vials using an 18G BD Blunt Fill™ filter needle (Becton, Dickinson and Co., Franklin Lakes, NJ, USA).

The procedures followed local standard protocol that included the use of 2–3 drops of topical tetracaine anaesthesia, use of an eye speculum, topical 5% povidone‐iodine disinfection, 33G needle (Zero Residual™ [SJJ Solutions BV, The Hague, The Netherlands] or TSK STERiJECT™ [TSK Laboratory, Tochigi, Japan]), injection site 3.5 mm posterior from the limbus marked by callipers in the superotemporal or the inferotemporal quadrant, sterile cotton tip tamponade at the site of injection after removal of the needle and no post‐procedure antibiotics (Petri et al., 2020).

Patients were counselled to report inflammatory symptoms promptly. Our department operates with a 24‐h telephone service, through which patients have easy access to see an ophthalmologist.

Routine follow‐up check‐ups were scheduled according to a local protocol where patients underwent visual acuity and optical coherence tomography examination, and depending on the diagnosis, they were seen by either a physician or a screening nurse, in which latter case, slit lamp examination was not done. In cases of symptoms hinting at inflammation, either at a routine checkup or reported through the telephone service, additional acute follow‐ups were scheduled with an ophthalmologist. If retinal vasculitis was suspected, wide‐field fluorescein and indocyanine‐green angiography were performed. Following the diagnosis of IOI, patients were surveyed for as long as they exhibited clinical signs and needed anti‐inflammatory treatment, then after the resolution of the IOI, they returned to routine follow‐up.

### 2.5 Data collection

The data collection was carried out by using the centre's electronic medical records database, injection database and by reviewing patient charts. Collected data for all participants included demographics (age, sex), indication for anti‐VEGF treatment (diagnosis), eye laterality, previous anti‐VEGF treatment history and total number of faricimab injections. Clinical diagnosis was done by extracting diagnosis codes based on the Danish Medical Coding Classification System, which uses the ICD10 system with national extensions (The Danish Health Data Authority, n.d.). Diagnosis codes were collected for each patient and filtered for relevance with regard to anti‐VEGF treatment and then grouped into the following categories: neovascular AMD (nAMD), central serous chorioretinopathy (CSC) with secondary choroidal neovascularization (CNV), other types of CNVs, central retinal vein occlusion (CRVO), branch retinal vein occlusion (BRVO), or DME. If grouping was not possible due to missing relevant diagnoses or in the presence of multiple contradicting ones, manual selection of the most relevant clinical diagnosis was carried out by two experienced retina specialists (FA, LT) from patient charts to ensure the correct classification of each patient.

Additional manual data extraction from patient charts was carried out by 3 experienced retina specialists (FA, LT, MS) for those diagnosed with IOI following faricimab injections. The extraction included number of faricimab injections after which the IOI manifested, date of the first faricimab injection, date and batch number of the faricimab injection preceding the IOI event, onset of symptoms, date of the diagnosis of IOI, latest pre‐onset best‐corrected visual acuity (BCVA), BCVA at the time of diagnosis of IOI, clinical characteristics of the IOI (presence of cells in the anterior chamber [AC], presence of keratic precipitates, hypopyon, fibrin, cells in the vitreous, vitreous haze or impaired view of the fundus, presence of retinal vasculitis, occlusive vasculitis, retinal infiltrates and/or haemorrhages, optic nerve head or macular oedema), results from culture and 16S ribosomal RNA PCR if a vitreous sample was obtained, type of inflammation (according to Standardization of Uveitis Nomenclature [SUN] classifications (Jabs et al., 2005)), type of action taken (topical anti‐inflammatory treatment, pars plana vitrectomy, intravitreal antibiotic, etc.), reasons for taking a particular action (if relevant), date of action(s), date the patient was declared free of inflammation, final BCVA at follow‐up, follow‐up time to determine the final functional outcome.

BCVA was recorded in Snellen and then converted to logarithm of the minimum angle of resolution (logMAR) units for analysis using the following formula:

$$\text{logMAR}=-\log_{10}\left(\text{Snellen fraction}\right)$$

Counting fingers, hand motion and light perception vision were converted to logMAR values of 1.9, 2.3 and 2.7, respectively, as per the report by Bach et al. (2007).

### 2.6 Outcome measures

The primary endpoint of the study was the incidence of IOI events following a faricimab injection procedure within the observation window, described as cases per eye, cases per patient and cases per injection.

Secondary endpoints included differences in patient characteristics between individuals developing and not developing IOI following faricimab injection, differences between treatment‐naïve and switched patients, time from injection to onset of IOI and clinical characteristics and functional outcomes of IOI cases.

### 2.7 Statistical analysis

Statistical analyses were conducted using Microsoft Excel 365 (version 2502, Microsoft Corp., Redmond, Washington, USA) and RStudio v. 2024.12.1 (RStudio Team, 2022) with R version 4.4.1 (R Core Team, 2022).

If an eye presented with a recurrence of IOI following a rechallenge with faricimab, only the first event was included in the calculations.

Confidence intervals (CIs) for groups with equal to or more than 5 exposures were calculated with the Wilson Score Interval; for groups with <5 exposures, the calculation was done by the Clopper‐Pearson Exact method.

According to inspection of distribution plots, no parameters in the IOI group were normally distributed; therefore, continuous variables were described with median and interquartile range (IQR) and compared using Mann–Whitney *U* test for unpaired samples and the Wilcoxon signed‐rank test for paired samples. Contingency tables were analysed by chi‐square test for sample sizes >5 and with Fisher's exact test for sample sizes ≤5.

Risk factor analysis for IOI was performed using Cox proportional hazards regression, with the number of faricimab injections serving as the underlying time scale. For eyes that developed IOI, the event time was defined as the injection at which the first IOI episode occurred. Eyes without IOI were right‐censored at their last recorded faricimab injection. Clinically relevant covariates were selected a priori and included age, sex, treatment‐naïve versus switched status and prior exposure to ranibizumab and aflibercept. In the primary multivariable model, diagnostic categories without IOI events were excluded to avoid separation; thus, the model was restricted to eyes with nAMD, CSC or DME, using nAMD as the reference category (restricted model). A secondary model including all eyes and omitting diagnosis was fitted as a robustness analysis (unrestricted model). Hazard ratios (HRs) with 95% CIs were reported. Assumptions of proportional hazards were evaluated using Schoenfeld residuals, with no meaningful violations observed. All time‐to‐event analyses were conducted in R using the survival package.

*p*‐values of <0.05 were considered statistically significant.

## 3 RESULTS

### 3.1 Demographics, prior injections and diagnoses

Our database search identified 6054 patients and 7510 eyes eligible for inclusion in this study within the selected 10‐month time period, and the search accounted for 31 393 injections. Of the eyes included, 49.4% (*n* = 3708) were right eyes and 50.6% (*n* = 3802) were left eyes. The median age of patients was 82 years (range: 24–106 years). There were 63.9% females (*n* = 3868) and 36.1% males (*n* = 2186).

The eyes included in this study were treatment‐naïve prior to receiving faricimab in 18.4% (*n* = 1384), whereas 78.0% (*n* = 5858) were switched from another anti‐VEGF treatment (switched eyes). In 268 eyes (3.6%), we were not able to extract information on previous treatments predating the first faricimab. Among the switched eyes, 99.6% (*n* = 5837) had received aflibercept, and 14.1% (*n* = 826) had received ranibizumab injections, and 13.7% (*n* = 805) had received both. The median numbers of aflibercept and ranibizumab were 16 (IQR: 25, range: 1–149) and 7 (IQR: 15, range: 1–116), respectively.

The primary indication for treatment was nAMD in the majority of eyes (88.3%, *n* = 6632), followed by other types of CNVs (such as myopic, peripapillary, inflammatory, or idiopathic), DME, CNV secondary to central serous chorioretinopathy (CSC), macular oedema due to RVO or other causes (Table 1).

**TABLE 1:** Incidence of intraocular inflammation in various categories.

Category	Total (*n*)	IOI (*n*)	Incidence %, (95% CI)
Patients	6054	40	0.66 (0.49–0.90) per patient
Eyes	7510	42	0.56 (0.41–0.76) per eye
Injections	31 393	42^a^	0.13 (0.10–0.18) per injection
Sex			
Males	2186	8	0.37 (0.19–0.72) per patient
Females	3868	32	0.83 (0.59–1.17) per patient
Prior treatments			
Switched eyes	5858	39	0.67 (0.49–0.91) per eye
Treatment‐naïve eyes	1384	3	0.22 (0.04–0.63) per eye
Missing data on prior treatments	268	–	
Diagnoses			
nAMD	6632	37	0.56 (0.41–0.77) per eye
CNV	228	0	0
DME	230	1	0.43 (0.01–2.40) per eye
CSC with secondary CNV	206	4	1.94 (0.53–4.90) per eye
BRVO	121	0	0
CRVO	90	0	0
Other (CME, neovascular glaucoma, PEHCR)	3	0	0

Abbreviations: BRVO, branch retinal vein occlusion; CI, confidence interval; Switched eyes: eyes previously treated with other anti‐VEGF drugs and switched to faricimab afterwards; CME, cystoid macular oedema; CNV, choroidal neovascularization due to causes other than AMD or CSC; CRVO, central retinal vein occlusion; CSC, central serous chorioretinopathy; DME, diabetic macular oedema; IOI, intraocular inflammation; nAMD, neovascular age‐related macular degeneration; PEHCR, peripheral exudative haemorrhagic chorioretinopathy.

^a^ Counting the first IOI episode for each eye only. Two patients had IOI in both eyes, of which one eye had a repeated episode; an additional 5 patients had a repeated IOI in one eye, resulting in a total of 48 IOI episodes.

### 3.2 Incidence of IOI and predictors

During the inclusion period, 42 eyes of 40 patients developed IOI, resulting in a total of 48 IOI events (2 patients had IOI in both eyes, of which one eye had a repeated episode; an additional 5 patients had a repeated IOI in one eye). As mentioned previously, only the first IOI for each eye was included in the incidence calculations, resulting in a total of 42 independent events.

The incidence of IOI was 0.66% per patient, 0.56% per eye and 0.13% per injection (Table 1).

There was a disproportionately high representation of females (80.0%, *n* = 32) among the patients with IOI (IOI incidence for females: 0.83%), and the Cox regression hazard models showed that females had a significantly higher hazard of IOI compared to males (restricted model: HR 2.66 [95% CI 1.22–5.81; *p* = 0.014], and unrestricted model: HR 2.42 [95% CI 1.12–5.24; *p* = 0.025]). Age showed no significant association with the occurrence of IOI (Table S1).

The eyes that developed IOI were switched from a previous anti‐VEGF in 92.9% (*n* = 39), while only 7.1% (*n* = 3) were treatment‐naïve, leading to a higher incidence rate (0.67%) among those that were switched versus those that were naïve (0.22%) (Table 1). In the Cox regression models, whether the patient had been switched from another anti‐VEGF agent or was treatment‐naïve was not significantly associated with IOI (*p* ≥ 0.642). The number of previous ranibizumab injections showed no significant associations either, and prior aflibercept exposure demonstrated a borderline association (HR 1.01 per injection; 95% CI 1.00–1.03 in both the restricted and unrestricted models; *p* ≥ 0.062) (Table S1).

We observed IOI in patients with primary diagnosis of nAMD, CSC with secondary CNV and DME only (Table 1). Among the diagnoses, CSC with secondary CNV had the highest IOI incidence rate (1.94% per eye, Table 1). In the restricted Cox regression model, CSC was also associated with a markedly increased hazard compared with AMD (HR 4.62; 95% CI 1.47–14.48; *p* = 0.009), whereas the estimate for DME was imprecise and non‐significant due to wide confidence intervals (Table S1).

### 3.3 Clinical characteristics

The eyes developing IOI were right eyes in 57.1% (*n* = 24) and left eyes in 42.9% (*n* = 18). Eyes that developed IOI had previously undergone cataract surgery in 59.5% (*n* = 25) of cases, none of which occurred within 6 months prior to the IOI.

IOI occurred after a median of 4 faricimab injections (IQR: 2, range: 1–9; for those with repeated episodes, only the first IOI event was included in the calculation).

Temporal metrics were analysed at the event level (*n* = 48). The onset of symptoms was retrievable in 40 cases, and it was unknown in 8 cases. The median time from the faricimab injection preceding the IOI event to the onset of symptoms was 8.5 days (IQR: 20, range: 0–37, *n* = 40), with a median time from the onset of symptoms to the clinical diagnosis of IOI being 2 days (IQR: 7, range: 0–31, *n* = 40). The median time from the last faricimab injection before the IOI to the diagnosis of IOI was 22 days (IQR: 22.25, range: 1–56), calculated in all 48 events. Following diagnosis, treatment was initiated promptly—the median time from diagnosis to treatment was 0 days (IQR: 0, range: 0–1, *n* = 48). The median disease duration (i.e., time from diagnosis to the resolution of IOI without need for further treatment) was 32 days (IQR: 46, range: 6–144, *n* = 46, missing data in 2 cases where the date of resolution was uncertain). The median total length of the follow‐up time was 38 days (IQR: 51.75, range: 6–144, *n* = 48). At the end of follow‐up, all IOI cases were accounted for, and none were lost to follow‐up.

Most IOI cases were anterior or anterior + intermediate uveitis (47.92% [*n* = 23] and 33.33% [*n* = 16], respectively), with 6.25% (*n* = 3) being intermediate uveitis and 12.50% (*n* = 6) panuveitis. For the distribution of IOI cases according to uveitis type, see Figure 1. Fluorescein and indocyanine‐green angiography were performed in 12 patients (30%), showing progressive hyperfluorescence of the optic nerve head with capillary leakage (hot disc) in 10 cases, without a sign of vascular occlusions, infiltrates or perivascular leakage elsewhere. In the other 2 cases, no abnormalities were seen. No cases of retinal vasculitis, occlusive vasculitis or retinal infiltrates were observed. All panuveitis cases were initially suspected to have endophthalmitis (per‐injection incidence: 0.019%, 95% CI: 0.01–0.04) and underwent vitrectomy. Five had negative microbiological examinations for bacteria, and one had a culture positive for *Staphylococcus epidermidis* (per injection incidence: 0.003%, 95% CI: 0.00–0.02).

**FIGURE 1:**
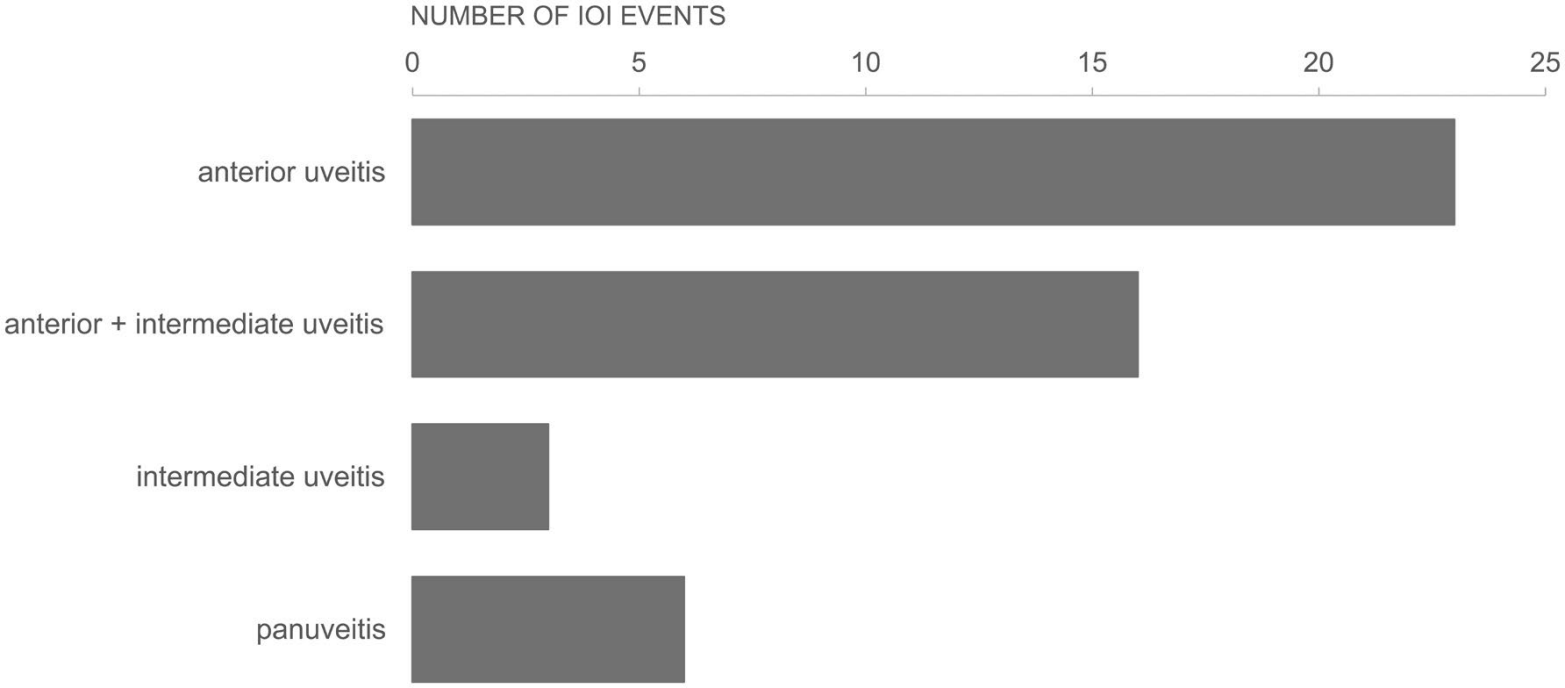
Number of cases with inflammation following faricimab injections broken down by the Standardization of Uveitis Nomenclature (SUN) anatomical classification.

Following the diagnosis of IOI, topical steroid treatment (dexamethasone 1%) was initiated in all cases (*n* = 48), and the majority of patients responded well. The treatment was supplemented with topical mydriatics (cyclopentolate 1%) in 20.83% (*n* = 10) of the cases and with IOP‐lowering agents in 16.67% (*n* = 8). Systemic treatment with corticosteroids was necessary in 10.42% (*n* = 5) and with oral valacyclovir (due to suspicion of herpetic co‐occurrence) in 4.17% (*n* = 2). In 7 cases (14.58%), pars plana vitrectomy was performed, and in the 6 cases (12.50%), which were initially suspected to be endophthalmitis, intravitreal antibiotics (vancomycin and ceftazidime) were administered following the vitrectomy, followed by topical dexamethasone.

Intraocular pressure (IOP) was elevated (>30 mmHg) in 7 cases at the diagnosis of the IOI, all resolved with topical treatment—2 cases needed dexamethasone only, and 5 required additional IOP‐lowering agents. IOP measurement at onset was not available in 1 case.

Following the resolution of the IOI, a decision to switch to aflibercept 2 mg was made in 54.76% of cases (*n* = 23) and in 14.29% (*n* = 6), the treatment was stopped. In 30.95% of cases (*n* = 13), faricimab treatment was rechallenged, and 46.15% of those (*n* = 6) relapsed (in 3 cases immediately after the next faricimab injection, in 2 cases after 3 injections and in 1 case after 4 injections). The presentations were consistent with the first event (*n* = 4) or less severe (*n* = 2). Three cases presented as anterior uveitis, 2 as anterior and intermediate uveitis and 1 case as intermediate uveitis. Event timing and disease duration were largely consistent for both initial IOI and recurrence. One case had an IOP increase, consistent with the first episode, and all cases had a final BCVA outcome consistent with the first episode (within ±1 Snellen line difference). These relapsed cases were subsequently switched to aflibercept 2 mg as well. The remaining 53.85% (*n* = 7) showed no relapse in the following 3 months or longer and continued on faricimab.

### 3.4 Differences between patient groups

After grouping the patients into two categories, one being those who developed IOI (IOI group, *n* = 40) and the other being those who did not develop IOI (control group, *n* = 6014), the median age was similar between groups, while the proportion of females was higher in the IOI group.

Regarding anti‐VEGF treatment, the median number of faricimab injections per eye was comparable between groups. However, eyes in the IOI group received more previous aflibercept injections per eye compared to controls, with the difference remaining significant when analysing only eyes that received at least one aflibercept injection. Regarding ranibizumab, most eyes in both groups received no injections, and among those that received ≥1 ranibizumab injection, the number of injections per eye did not differ significantly between the groups.

AMD was the most prevalent condition in both groups, where it exhibited a comparable distribution. However, CSC with secondary CNV was more frequent in the IOI group. One eye with DME had IOI, and no other diagnoses were present in the IOI group. The proportion of treatment‐naïve eyes was lower in the IOI group, with most eyes having previously received other anti‐VEGF treatments.

For a detailed comparison between the control and IOI groups, see Table S2.

### 3.5 Functional outcomes

The median BCVA before the onset of the IOI was 0.30 logMAR (IQR: 0.36). At diagnosis, BCVA dropped to a median of 0.7 logMAR (IQR: 0.70), and at the end of follow‐up, it increased to 0.4 logMAR (IQR: 0.48). A statistically significant difference was found between the pre‐onset values and those measured at diagnosis of IOI, as well as between the latter values and follow‐up (*p* < 0.001 for both). However, there was no significant difference between the values at pre‐onset and follow‐up (*p* = 0.088). Severe visual loss equivalent to 2 or more Snellen lines at final follow‐up compared to pre‐onset levels was observed in 11 cases. There were missing data for one observation before IOI, 3 observations at the diagnosis of IOI and 1 observation at the end of follow‐up. For the development of BCVA values, see Figure 2.

**FIGURE 2:**
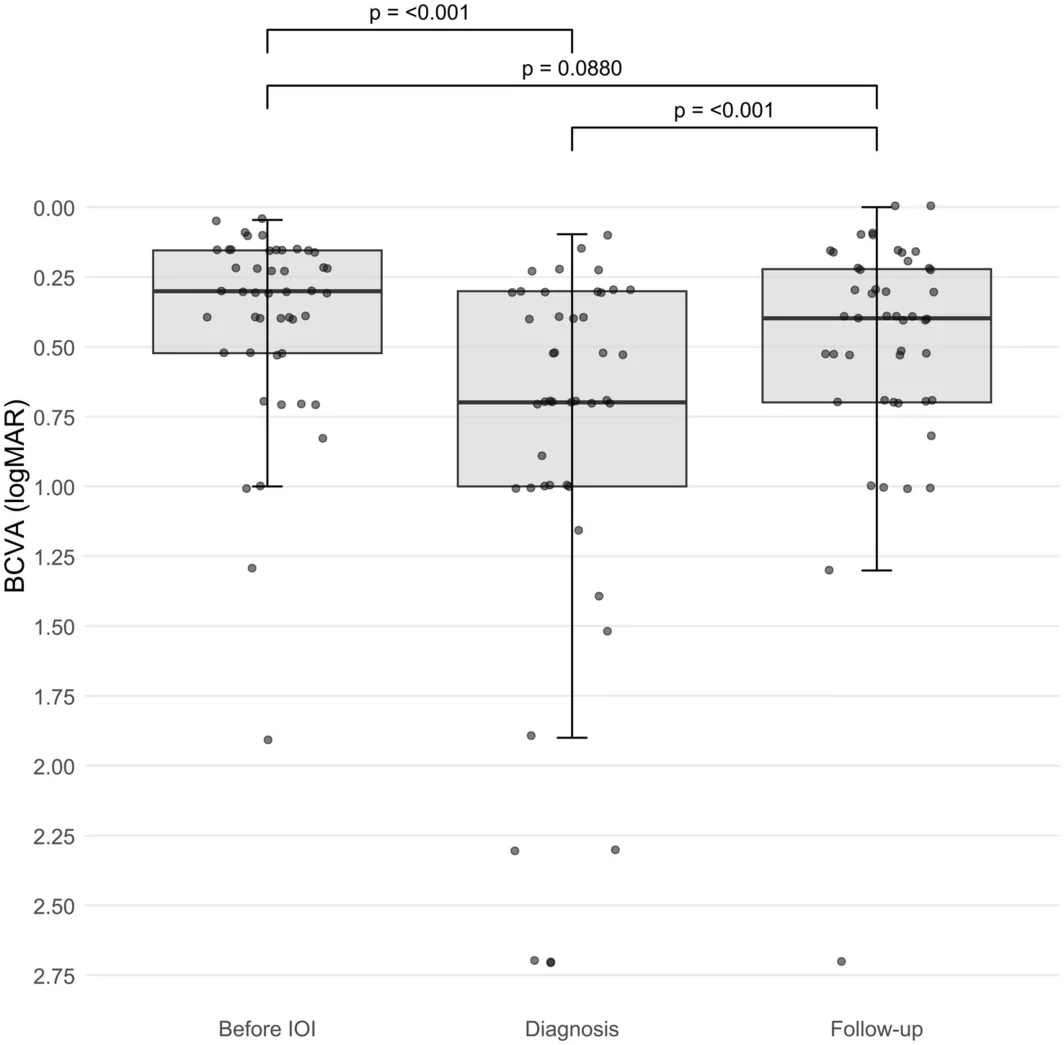
Boxplots of the distribution of best‐corrected visual acuity at the last examination before the intraocular inflammation (IOI), at the diagnosis of IOI, and at the final follow‐up after IOI was resolved.

## 4 DISCUSSION

This study focused on reporting real‐world safety data on IOI following faricimab treatment using a large dataset from a highly specialized tertiary centre in Denmark.

Intravitreal anti‐VEGF injections represent a cornerstone of the treatment of numerous retinal diseases, yet they are associated with several inflammatory adverse events that warrant careful consideration. Real‐world data from heterogeneous patient groups provide important insights into these side effects that complement the findings of clinical trials. This helps create more detailed approaches to patient management. Understanding inflammatory complications in different clinical settings is crucial for optimizing treatment and improving visual outcomes for patients receiving anti‐VEGF therapy.

According to our knowledge, this is the largest single‐centre real‐world safety dataset on faricimab to date. Globally, it is surpassed in size only by multi‐centre real‐world studies such as the FARWIDE‐DMO (Peto et al., 2025) and FARETINA‐AMD (Tabano et al., 2024), which draw data from broader patient registries.

The incidence of IOI in the registration trials ranged from 0.4% to 2.2% in their respective faricimab arms (Heier et al., 2022; Tadayoni et al., 2024; Wykoff et al., 2022). Real‐world studies available to date typically find somewhat lower rates of inflammation. These differences can be attributed to the frequency of monitoring, data capture and reporting. Most of our results are consistent with the other real‐world studies. The FARETINA‐AMD/DME studies (Tabano et al., 2024) that involved 26 278 AMD and 6343 DME patients and approximately 276 246 injections, reported a per‐injection incidence of 0.12% and 0.1% of IOI in treatment‐naïve and previously treated groups, respectively. Other real‐world studies, such as the TRUCKEE (Khanani et al., 2023) and the FARWIDE‐DMO (Peto et al., 2025), a recent report from the Wills Eye Hospital (Momenaei et al., 2024), or the Moorfields Eye Hospital (Montesel et al., 2025) report similar results with IOI incidences ranging from 0.14% to 0.7% per injection. Generally, these figures align well with our experiences, as we found a 0.13% per‐injection incidence of IOI for the whole cohort.

Neither the FARETINA (Tabano et al., 2024) nor the Moorfields (Montesel et al., 2025) study found variations in the incidence of IOI between groups with different treatment histories or primary indications. In our study, however, eyes previously treated with other anti‐VEGF drugs had 3 times higher incidence of developing IOI following treatment with faricimab than treatment‐naïve eyes. In particular, previous aflibercept exposure led to borderline increased hazard of IOI following faricimab, which is probably due to their molecular similarities. Both aflibercept and faricimab have a human IgG1 Fc portion, while ranibizumab consists solely of a Fab fragment (Fogli et al., 2018; Liberski et al., 2022). Switched patients often have extensive exposure to previous anti‐VEGF agents; in our cohort, the number of prior aflibercept and ranibizumab injections in those switched to faricimab was up to 149 and 116, respectively. This exposure could lead to the formation of anti‐drug antibodies (ADA) (Sharma et al., 2019) that might recognize epitopes in faricimab. Besides treatment‐emergent ADAs, pre‐existing ADAs might also play a role (Kim & Woo, 2024). Additionally, chronic VEGF suppression might maintain a low‐grade inflammation and alter the ocular immune tolerance, as well as lead to the upregulation of inflammatory cytokines (Sato et al., 2021).

Another interesting observation in our study was that the primary indication of CSC with secondary CNV yielded almost four times higher IOI incidence, with a 4.6 times increased hazard compared to nAMD. However, the low number of such patients in our IOI cohort prevents meaningful conclusions; the higher incidence could potentially point to disease‐specific vulnerabilities in CSC. The pathology of CSC is characterized by choroidal vascular congestion, hyperpermeability and subclinical inflammation (Bousquet et al., 2023; Feenstra et al., 2024; Subhi et al., 2019). Anti‐VEGF therapy may disrupt compensatory VEGF‐mediated vascular stabilization in an already stressed choroid. Additionally, choroidal vessels rely on VEGF for endothelial maintenance (Marneros et al., 2005). An anti‐VEGF‐induced depletion could exacerbate choroidal ischemia and lead to a hypoxia‐driven inflammation. Damaged RPE and breakdown of the blood‐retina barrier in CSC might also play a role by exposing retinal antigens to immune cells. The unique choroidal pathophysiology of CSC and its reliance on VEGF for vascular homeostasis, along with the pre‐existing inflammatory milieu, could, in theory, create a perfect storm for inflammation when these pathways are inhibited, leading to cumulative insults to an already compromised system. However, these theories need to be investigated through comparative studies with both CSC and AMD cohorts.

We also found that females had more than twice as high an incidence of IOI and a two‐ to threefold increased hazard compared to males. Some previous studies have reported that female sex may be associated with a higher risk of developing IOI after certain anti‐VEGF treatments, particularly brolucizumab. A Japanese multi‐centre study found that females were nearly twice as likely to develop IOI following brolucizumab treatment, with an odds ratio of 1.99 (Inoda et al., 2024). Witkin et al. also described a female predominance of retinal vasculitis associated with brolucizumab, which may suggest an autoimmune response, though no definitive link to autoimmune disease has been established (Witkin et al., 2020). Conversely, a South Korean study associated female sex with a decreased risk of clinically significant IOI, showing a hazard ratio of 0.78 (Park et al., 2024). This suggests a complex relationship that may vary by population or study design. According to our knowledge, this phenomenon of female predominance was not yet described in association with faricimab in real‐world studies, but a recent multi‐source pharmacovigilance analysis by Liu et al. (2025) corroborates this finding.

The median number of faricimab injections required to trigger the inflammation was 4 in our study, ranging from 1 to 9, and the median time from injection to symptoms (where these data were available) was 8.5 days. These data are consistent with existing real‐world literature (Cozzi et al., 2024; Momenaei et al., 2024; Montesel et al., 2025). The time from injection to diagnosis was somewhat longer, a median of 22 days with a broad range of 1–56 days, indicating that some patients may have experienced slowly developing, mild, or non‐specific symptoms before reporting to our department. Similar data have been reported by Cozzi et al. (2024). The delayed onset and the typical occurrence after repeated injections suggest a hypersensitivity reaction rather than acute toxicity (Anderson et al., 2021). Drug aggregate formation might also increase the risk of activating the immune system (Lundahl et al., 2021).

Non‐infectious IOI typically presents with AC reaction and vitritis, with most cases resolving after topical steroid therapy without permanent visual sequelae (Anderson et al., 2021). This was also our experience with this study, in which about half of the cases were anterior uveitis and one‐third were combined anterior and intermediate uveitis. They generally responded well to dexamethasone eyedrops and resolved over 1–20 weeks with good visual recovery. We observed a handful of more serious cases of inflammation where diagnostic vitrectomy was necessary to distinguish them from infectious endophthalmitis, and one tested positive for bacteria. There are several reports in the literature of severe IOI (Thangamathesvaran et al., 2024), including retinal vasculitis (Ben Ghezala et al., 2025) or occlusive vasculitis (Bruening et al., 2025; Cozzi et al., 2024; Li et al., 2024), but those cases are considered rare. We did not observe any vasculitis cases in our cohort, but we did not routinely perform angiography in all cases, which is a limitation.

Following the resolution of IOI, we switched the majority of our patients to an alternative anti‐VEGF. Among those cases where we chose to continue on faricimab after the IOI, we observed a relatively high relapse rate of 46%, and these were also switched to a different anti‐VEGF afterwards. Bruening et al. documented a case series in which three out of four eyes experienced severe and irreversible vision loss due to occlusive vasculitis after being rechallenged with faricimab injection following an initial mild AC reaction (Bruening et al., 2025). The authors postulated an immunological memory response that is triggered with repeated exposures. Fortunately, among our cases that were rechallenged and subsequently relapsed, we did not observe worse outcomes, and the repeated events showed largely consistent phenotype, disease course and visual outcome to the previous event. We agree, however, with Bruening et al. (2025) that caution is necessary when reusing the same biologic after an IOI from a previous injection.

Limitations of our study should be acknowledged. Although the strength of our study is the structured data collection in our databases and the ability to extract detailed patient characteristics and conduct a more granular analysis, the retrospective nature of the study comes with inherent biases. Additionally, at our centre, slit‐lamp eye examination is not performed routinely on each patient at each visit; therefore, subclinical or asymptomatic cases may have been overlooked, and the incidence of IOI may be underestimated. This might also explain the discrepancy between the data from the registration trials and real‐world experiences. It may be argued, however, that symptomatic IOI was captured and that the indication for treating asymptomatic low‐grade inflammation is uncertain. Also, some of our primary indications for initiating anti‐VEGF treatment were off‐label (such as CNV secondary to causes other than nAMD), which could limit the applicability of our results in some countries. Furthermore, the majority of our patients during the study period received treatment with pharmacy‐prepared pre‐filled syringes, while only a small number of doses were drawn directly from original vials in the injection rooms. This distribution limited our ability to make a meaningful comparison between these two groups. Additionally, we did not systematically collect data on IOI with other anti‐VEGF agents as comparators during the same period, which prevents direct comparisons. Another limitation of this study is the diagnostic challenge in distinguishing endophthalmitis from IOI. To ensure consistency in case classification, all cases initially suspected or confirmed as endophthalmitis were categorized as IOI, including the single case that yielded a positive bacterial culture. Lastly, ethnicity data, systemic inflammatory comorbidities and uveitis history were not collected; therefore, potential associations with IOI could not be explored.

In conclusion, faricimab demonstrated an acceptable safety profile in our study. The rate of IOI was at an acceptable low level; the incidence of IOI events was consistent with those observed in other real‐world reports. Eyes previously treated with aflibercept had a borderline increased hazard of IOI following faricimab. The IOI events had a female predominance; they showed varied presentations and durations, typically responded well to topical steroid treatment, and generally had favourable outcomes. Additionally, those who were rechallenged with faricimab following an initial IOI episode had a relatively high rate of relapse. As the therapeutic landscape continues to evolve with the introduction of newer agents like faricimab, ongoing surveillance and documentation of inflammatory complications in real‐world settings remain essential for optimizing patient safety and treatment efficacy.

## AUTHOR CONTRIBUTIONS

M.S.: Conceptualization; Methodology; Investigation; Data curation; Formal analysis; Validation; Visualization; Project administration; Supervision; Roles/Writing – original draft; Writing – review & editing. L.T.: Investigation; Data curation; Writing – review & editing. F.A.: Investigation; Data curation; Writing – review & editing. J.B.: Data curation; Formal analysis; Validation; Visualization; Writing – review & editing. O.N.K.: Conceptualization; Methodology; Writing – review & editing. C.F.: Conceptualization; Methodology; Writing – review & editing. Y.S.: Conceptualization; Methodology; Writing – review & editing. J.N.H.: Conceptualization; Methodology; Resources; Writing – review & editing. All authors read and approved the final manuscript. All authors agreed to be accountable for all aspects of the work in ensuring that questions related to the accuracy or integrity of any part of the work are appropriately investigated and resolved.

## FUNDING INFORMATION

None.

## CONFLICT OF INTEREST STATEMENT

M.S. declares to have served as an advisory board member for Novartis, Roche and Bayer, acted as a consultant for AbbVie, received speaker fees from AbbVie and Roche, and received travel grants from AbbVie, Bayer and Roche, not related to this work. Y.S. declares to have received speaker fees from Bayer and Roche, not related to this work. L.T., F.A., J.B., O.N.K., C.F. and J.N.H. declare that no potential conflicts of interest exist in relation to this work.

## ETHICS STATEMENT

The research and data handling were conducted in compliance with local laws and GDPR, the tenets of the Declaration of Helsinki and its amendments. The Regional Health Research Ethics Committees in the Capital Region in Denmark waived the need for ethical approval of the project (no. F‐24041134). The Research and Health Legal Department of the Capital Region of Denmark has approved the patient confidentiality and data flow protocol of the project (approval n.: p‐2024‐16902, date of decision: 04/11/2024) and waived the need for informed consent to access individual participant health data and to publish. The study protocol was registered at ClinicalTrials.gov, registration number: NCT06902207, date of registration: 15/03/2025.

## USE OF AN AI STATEMENT

The authors declare the use of generative artificial intelligence systems. Perplexity (Perplexity AI, San Francisco, CA, USA) and Copilot (Microsoft Corp., Redmond, Washington, USA) were used to improve the clarity of the text in the Introduction and Discussion sections. SciSpace (PubGenius Inc., Milpitas, CA, USA) was used to assist in literature research. All generated content was thoroughly checked for accuracy. The authors take responsibility for the integrity of the content generated.

## Supporting information


**Table S1.** Cox proportional hazards regression analysis for intraocular inflammation risk factors.

**Table S2.** Demographics of the control and IOI groups.

## Data Availability

Raw data sharing with third parties was not approved for this project. Data are located in controlled access data storage at the Capital Region of Denmark.
