# Supplementary material for: Real‐world incidence and clinical characteristics of intraocular inflammation following intravitreal faricimab therapy: A safety study
Source: Acta Ophthalmol. 2026 May 16;104(8):e1008–17. doi: 10.1111/aos.70161 (PMC13751878; doi:10.1111/aos.70161)
Supplement: Supplementary file 1 [file AOS-104-e1008-s001.docx]

**Supplementary table 1.** **Cox proportional hazards regression analysis for intraocular inflammation risk factors**

**Model 1 (restricted model):** Cox regression hazard ratio restricted to diagnoses of IOI cases (nAMD, CSC and DME; n= 6959, number of events= 42, 109 observations deleted due to missingness)

| Variable | Hazard ratio (95% CI) | p-value |
| --- | --- | --- |
| Age (per year) | 1.01 (0.97–1.05) | 0.745 |
| Sex: Female vs male | **2.66** (1.22–5.81) | **0.014** |
| Switch vs naïve | 1.27 (0.37–4.45) | 0.703 |
| Prior ranibizumab (per injection) | 0.98 (0.93–1.04) | 0.532 |
| Prior aflibercept (per injection) | 1.01 (1.00–1.03) | 0.062 |
| CSC vs nAMD | **4.62** (1.47–14.48) | **0.009** |
| DME vs nAMD | 4.56 (0.57–36.42) | 0.152 |

**Model 2 (unrestricted model):** Cox regression hazard ratio without diagnoses (n= 7242, number of IOI events= 42, 268 observations deleted due to missingness)

| Variable | Hazard ratio (95% CI) | p-value |
| --- | --- | --- |
| Age (per year) | 0.99 (0.96–1.03) | 0.755 |
| Sex: Female vs male | **2.42** (1.12–5.24) | **0.025** |
| Switch vs naïve | 1.34 (0.39–4.68) | 0.642 |
| Prior ranibizumab (per injection) | 0.98 (0.93–1.04) | 0.588 |
| Prior aflibercept (per injection) | 1.01 (1.00–1.03) | 0.066 |

Abbreviations: CI: Confidence interval; CSC: central serous chorioretinopathy; DME: diabetic macular edema; nAMD: neovascular age-related macular degeneration.

P-values and hazard ratios of statistically significant factors marked with **bold**.
