# Supplementary material for: Real‐world incidence and clinical characteristics of intraocular inflammation following intravitreal faricimab therapy: A safety study
Source: Acta Ophthalmol. 2026 May 16;104(8):e1008–17. doi: 10.1111/aos.70161 (PMC13751878; doi:10.1111/aos.70161)
Supplement: Supplementary file 2 [file AOS-104-e1008-s002.docx]

**Supplementary table 2.** **Demographics of control group and IOI group**

| Variable |  | Control group | IOI group | Group comparison p-value |
| --- | --- | --- | --- | --- |
| Eyes | n | 7468 | 42 |  |
| Patients | n | 6014 | 40 |  |
| Age | median [IQR] | 82 [10] | 82 [11.3] | 0.466 |
| Sex (Female) | n (%) | 3836 (63.8) | 32 (80.0) | **0.033** |
| Faricimab | Total injections, n | 31224 | 169 |  |
|  | Injections per eye, median [IQR] | 4 [3] | 4 [2] | 0.570 |
| Aflibercept | Total injections, n | 126461 | 1150 |  |
|  | All eyes:   Injections per eye, median [IQR] | 12 [24] | 22 [37] | **0.004** |
|  | Eyes with ≥1 injection(s):   Injections per eye, median [IQR] | 16 [25] | 25 [32] | **0.023** |
| Ranibizumab | Total injections, n | 10271 | 68 |  |
|  | All eyes:  Injections per eye, median [IQR] (range) | 0 [0] (0–116) | 0 [0] (0–24) | 0.132 |
|  | Eyes with ≥1 injection(s):   Injections per eye, median [IQR] | 7.5 [15] | 5 [6.3] | 0.563 |
| Diagnosis | nAMD, n (%) | 6595 (87.8) | 37 (88.1) | 1.000 |
|  | CNV, n (%) | 228 (3.1) | 0 (0.0) | - |
|  | DME, n (%) | 229 (3.0) | 1 (2.4) | 1.000 |
|  | CSC with secondary CNV, n (%) | 202 (2.7) | 4 (9.5) | **0.026** |
|  | BRVO, n (%) | 121 (1.6) | 0 (0.0) | - |
|  | CRVO, n (%) | 90 (1.2) | 0 (0.0) | - |
|  | Others (CME, neovascular glaucoma, PEHCR), n (%) | 3 (0.0) | 0 (0.0) | - |
| Switched or Treatment-naïve eyes | Treatment-naïve, n (%) | 1381 (18.4) | 3 (7.1) | 0.070 |
|  | Switched, n (%) | 5819 (77.5) | 39 (92.9) | **0.015** |
|  | Missing data | 268 | 0 |  |

Abbreviations: IOI: Intraocular inflammation; IQR: Interquartile range; nAMD: neovascular age-related macular degeneration; CSC: central serous chorioretinopathy; DME: diabetic macular edema; CNV: choroidal neovascularization; BRVO: branch retinal vein occlusion; CRVO: central retinal vein occlusion; CME: cystoid macular edema; PEHCR: peripheral exudative hemorrhagic chorioretinopathy.

P-values of statistically significant factors marked with **bold**.
